# Antibacterial Activity of *Rhodomyrtus tomentosa* (Aiton) Hassk. Leaf Extract against Clinical Isolates of *Streptococcus pyogenes*

**DOI:** 10.1155/2012/697183

**Published:** 2012-09-02

**Authors:** Surasak Limsuwan, Oliver Kayser, Supayang Piyawan Voravuthikunchai

**Affiliations:** ^1^Faculty of Traditional Thai Medicine and Natural Products Research Center, Faculty of Science, Prince of Songkla University, Hat Yai, Songkhla 90112, Thailand; ^2^Department of Bio- and Chemical Engineering, Technical University of Dortmund, Technical Biochemistry, Emil-Figge-Strasse 66-68, 44227 Dortmund, Germany; ^3^Department of Microbiology and Natural Products Research Center, Faculty of Science, Prince of Songkla University, Hat Yai, Songkhla 90112, Thailand

## Abstract

Ethanol extract of *Rhodomyrtus tomentosa* (Aiton) Hassk. leaf was evaluated for antibacterial activity against 47 clinical isolates of *Streptococcus pyogenes*. The extract exhibited good anti-*S. pyogenes* activity against all the tested isolates with similar minimum inhibitory concentration (MIC, 3.91–62.5 **μ**g mL^−1^) and minimum bactericidal concentration (MBC, 3.91–62.5 **μ**g mL^−1^) ranges. No surviving cells were detected at 16 h after treatment with 8 × MIC of the extract. The extract-treated cells demonstrated no lysis and cytoplasmic leakage through the bacterial membrane. Electron micrographs further revealed that the extract did not cause any dramatic changes on the treated cells. Rhodomyrtone, an isolated compound, exhibited good anti-*S. pyogenes* activity (14 isolates), expressed very low MIC (0.39–1.56 **μ**g mL^−1^) and MBC (0.39-1.56 **μ**g mL^−1^) values. *Rhodomyrtus tomentosa* leaf extract and rhodomyrtone displayed promising antibacterial activity against clinical isolates of *S. pyogenes*.

## 1. Introduction

*Streptococcus pyogenes*, also known as group A streptococcus, is a major upper respiratory tract bacterial pathogen that causes a wide variety of diseases from common and mostly uncomplicated cases of pharyngitis and impetigo to severe invasive infections [1]. It is the most common cause of bacterial pharyngitis in children and may lead to nonsuppurative complications, such as rheumatic fever and glomerulonephritis. Penicillin remains the treatment of choice for *S. pyogenes* infections based on its narrow antibacterial spectrum, good efficacy, safety profile, and low cost [2]. However, increasing failure rates (20–30%) of penicillin therapy for *S. pyogenes* infections have been reported by many controlled studies [3–8]. Some studies have even indicated failure rates of 40% to 80% with the second course of the treatment [4, 5]. Because of these increasing failure rates, questions arise whether or not penicillin still should be considered standard therapy [9]. Erythromycin and related macrolide antibiotics, which are used in patients with a known or suspected allergy to penicillin, are considered as alternative drugs [2, 10, 11]. Unfortunately, an increasing incidence of erythromycin resistance has been reported in several parts of the world in recent years [12–15]. Moreover, no vaccines are now available to prevent streptococcal infections and their sequelae. *Streptococcus pyogenes* vaccines are currently in development [16, 17]. Therefore, the discovery of potential new drugs might be helpful for the treatment of *S. pyogenes* infections in the near future.

 Many medicinal plants have been studied and some have a strong activity and good potential to be developed into an effective drug. Downy rose myrtle, *Rhodomyrtus tomentosa* (Aiton) Hassk., is a Thai medicinal plant used to treat oral, gastrointestinal, urinary tract infections, and used as an antiseptic wash for wounds. Our preliminary antibacterial screening data from a number of plants found that *R. tomentosa *leaf extract was very effective against many Gram-positive bacteria [18]. Therefore, the aims of this study were to evaluate this effective plant against various clinical isolates of *S. pyogenes *and examine its mechanisms of action.

## 2. Materials and Methods

### 2.1. Plant Extraction

Classified reference voucher specimen of *R. tomentosa* (NPRC0057) was deposited at Faculty of Traditional Thai Medicine, Prince of Songkla University, Thailand. The crude ethanol extract of *R. tomentosa* leaf and rhodomyrtone were prepared according to the previously published methodology [18]. The extract and compound were checked for the same qualitative and quantitative profiles that were comparable with recently published data [19]. They were dissolved in 100% dimethyl sulfoxide (DMSO, Merck, Germany) before use (10 g L^−1^for the crude extract and 1 g L^−1^ for rhodomyrtone).

### 2.2. Bacterial Strains and Culture Conditions

Forty-seven clinical isolates of *S. pyogenes* (NPRC 101-147) were obtained from patients admitted at Prince of Songkla Hospital with tonsillitis or pharyngitis. A throat swab of each patient was individually plated onto Columbia blood agar base (Oxoid, UK) containing 5% sheep red blood cells (BA). Betahaemolytic streptococcallike colonies were subjected to appropriate biochemical testing as described previously [20]. A reference strain, *S. pyogenes* DSM 11728, was obtained from the German Collection of Microorganisms and Cell Cultures (DSMZ, Braunschweig, Germany). The bacterial cultures were stored in brain heart infusion (BHI) broth (Difco, France) containing 20% glycerol at −70°C until use. All isolates were cultured on BA plates incubated with 5% CO_2_ at 37°C for 24 h.

### 2.3. Antibacterial Activities

Broth microdilution method was carried out according to Clinical and Laboratory Standards Institute Guidelines [21]. Minimum inhibitory concentration (MIC) was recorded as the lowest concentration that produced a complete suppression of visible growth. An aliquot (20 *μ*L) from the broth (200 *μ*L) with no growth were pipetted and dropped onto BA plates and incubated with 5% CO_2_ at 37°C for 24 h. The minimum bactericidal concentration (MBC) was defined as the lowest concentration of the extract completely preventing bacterial growth. Penicillin G and erythromycin (Sigma, France) were used as reference antibiotics. 1% DMSO was used as a negative control. All tests were performed in triplicate independent experiments.

### 2.4. Time-Kill Assay

The bactericidal activity of the extract was studied using a time-kill assay [22]. A representative isolate *S. pyogenes* NPRC 101 was used in this study. Suspension of *S. pyogenes *in 0.85% normal saline solution (NSS) at the stationary phase of growth was prepared from the culture on BHI agar. The bacterial suspension was added to BHI broth containing the extract at 1/2 × MIC, MIC, 2 × MIC, 4 × MIC, and 8 × MIC and incubated with 5% CO_2_ at 37°C. The final cell concentration was 5 × 10^5^ CFU mL^−1^. The samples were collected at 2 h intervals over 24 h period, and the surviving bacteria were cultured on BA. 1% DMSO was used as a negative control. The assay was carried out in duplicate.

### 2.5. Bacteriolysis

A modified method from Carson et al. [23] was used in this experiment. Briefly, suspensions of *S. pyogenes* NPRC 101 in NSS were prepared from the culture on BHI agar. The suspensions were supplemented with the plant extract at 1/2 × MIC, MIC, 2 × MIC, and 4 × MIC and mixed with a vortex mixer. The final cell concentration was 1.5 × 10^8^ CFU mL^−1^. Optical density at 620 nm (OD620) was measured at 2 h intervals until 24 h to detect cell lysis as indicated by a decrease in OD620. Corresponding dilutions of test agents were used as blank, and 1% DMSO was used as a negative control. The assay was carried out in triplicate.

The results were expressed in percent as the ratio of OD620 at each time interval versus OD620 at 0 min.

### 2.6. Loss of 260-nm-Absorbing Materials

A modified method from Carson et al. [23] was used in this assay. Suspension of *S. pyogenes* was prepared from the culture on BHI agar. The bacterial cells were washed twice with NSS and resuspended in NSS. The extract was added at final concentrations equivalent to 1/2 × MIC, MIC, 2 × MIC, and 4 × MIC. The final cell concentration was 1.5 × 10^8^ CFU mL^−1^. 1% DMSO was used as a negative control. Samples were removed at 0, 2, 4, 6, 8, 10, 12, and 24 h, diluted 1 in 100, filtered through a 0.2 *μ*m pore-size filter, and OD260 was determined. Filtrates of appropriate dilution of each agent were prepared and used as blank. OD260 at each time point was expressed as a proportion of initial OD260. The assay was carried out in triplicate. Mean ratios for each treatment extract and time were calculated and compared to the means for the corresponding untreated samples. A Dunnett-ANOVA test was used to compare between the tests and control at each time point.

### 2.7. Transmission Electron Microscopy

The bacterial suspension of *S. pyogenes* in NSS (1.5 × 10^9^ CFU mL^−1^) was prepared from the culture on BHI agar. Then, 1 mL of suspension was added into 9 mL BHI broth supplemented with the extract at its MBC. The suspensions were incubated with 5% CO_2_ at 37°C for 14 h according to the time before the cells were killed with the extract. The bacterial cells were collected and prepared for transmission electron microscopy [24].

## 3. Results

### 3.1. Antibacterial Activities

The antibacterial activities of the ethanol extract of *R. tomentosa* and reference antibiotics against 47 clinical isolates of *S. pyogenes *were expressed as MIC and MBC (Table 1). All strains tested with penicillin (MIC ≤ 0.12 *μ*g mL^−1^) and erythromycin (MIC ≤ 0.25 *μ*g mL^−1^) were sensitive to both antibiotics. The extract of *R. tomentosa* showed significant activity against all 47 clinical isolates with similar MIC (3.91–62.5 *μ*g mL^−1^) and MBC (3.91–62.5 *μ*g mL^−1^) ranges. The MIC_50_ and MIC_90_ of the extract on *S. pyogenes* were 7.81 and 15.62 *μ*g mL^−1^, respectively. The antibacterial activity of rhodomyrtone was evaluated against 14 clinical isolates of *S. pyogenes.* It exhibited good anti-*S. pyogenes* activity, with very low MIC (0.39–1.56 *μ*g mL^−1^) and MBC (0.39–1.56 *μ*g mL^−1^) values. The MIC_50_ and MIC_90_ were 0.78 and 1.56 *μ*g mL^−1^, respectively.

### 3.2. Time-Kill Assay

**Figure 1:**
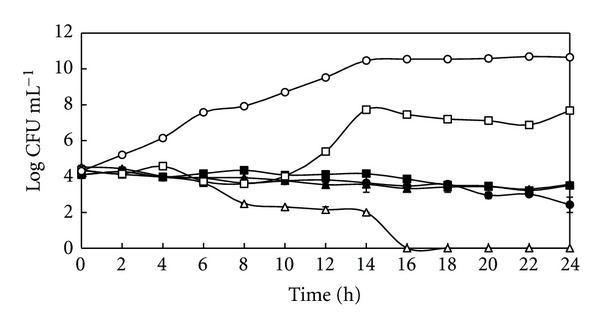
Time-kill curves of *Streptococcus pyogenes* NPRC 101 after treatment with the ethanol extract of *Rhodomyrtus tomentosa *leaf at 1/2 × MIC (□), MIC (■), 2 × MIC (▲), 4 × MIC (●), and 8 × MIC (∆). 1% DMSO was used as a control (○). Each symbol indicates the mean ± SD.

Time-kill curves of *S. pyogenes* after treatment with the plant extract are demonstrated in Figure 1. *Rhodomyrtus tomentosa* extract exhibited killing effect at 8 × MIC (MBC) after 16 h, while the concentration at 4 × MIC and 2 × MIC inhibited growth of the organisms. At 1/2 × MIC, the extract inhibited the bacterial growth until 10 h, reached log phase and stationary phase after 10 and 14 h, respectively. The growth level at the stationary phase was 2-3 log CFU mL^−1^ lower than the control until 24 h.

### 3.3. Bacteriolysis

**Figure 2:**
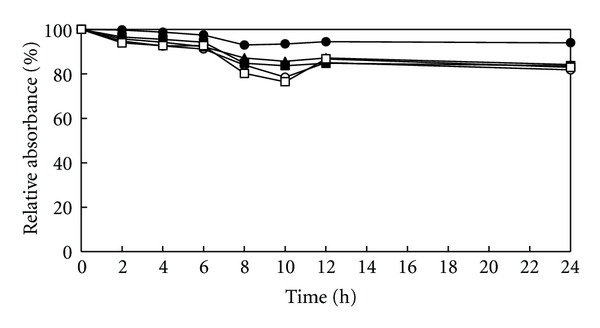
Bacteriolytic activity of the ethanol extract of *Rhodomyrtus tomentosa *leaf against *Streptococcus pyogenes* NPRC 101 at 1/2 × MIC (□), MIC (■), 2 × MIC (▲), 4 × MIC (○), and 1% DMSO (●). The results were expressed in percent as the ratio of OD620 at each time interval versus OD620 at 0 min (relative absorbance). The values are from triplicate experiments.

The bacteriolytic activity of the extract on *S. pyogenes* is presented in Figure 2. Treatment of *S. pyogens* cells with the extract at all concentrations produced no effect on bacterial cell lysis within 24 h (% relative absorbance at OD620 range from 80.2–99.7%).

### 3.4. Loss of 260-nm-Absorbing Materials

**Figure 3:**
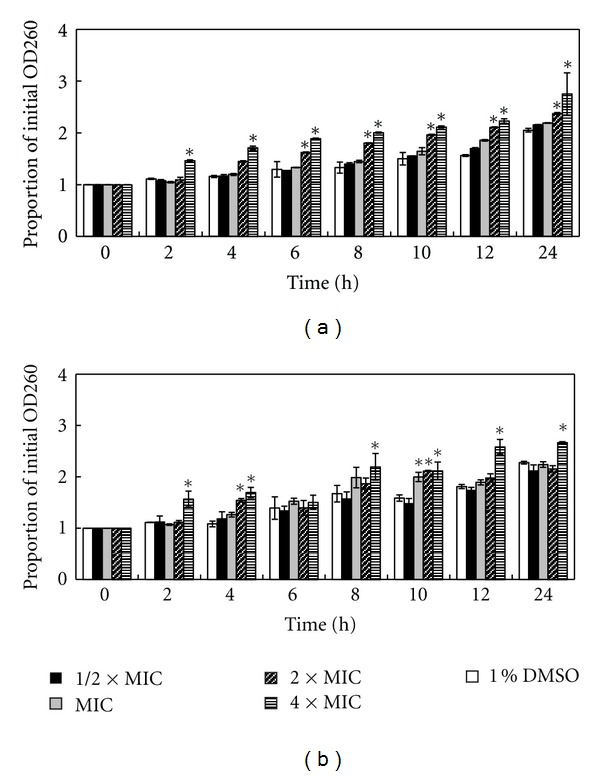
Measuring absorbance of the cell material contents at 260 nm releasing from *Streptococcus pyogenes* NPRC 101 (a) and *S. pyogenes* NPRC 109 (b) after treatment with the ethanol extract of *Rhodomyrtus tomentosa* leaf at 1/2 × MIC, MIC, 2 × MIC, 4 × MIC, and 1% DMSO. Mean values of triplicate independent experiments and SDs are shown. Dunnett test demonstrates significant difference between the tests and the control at each time point (*P < 0.05).

Leakage through bacterial cytoplasmic membrane was analysed by determining of the absorbance reading at 260 nm (Figure 3). The results demonstrated that only the filtrate from 4 × MIC of the treatment with *R. tomentosa* extract was significantly different from the control after 2 h (*P* < 0.05). Significant increase in the OD260 s occurred at 2 × MIC and 4 × MIC during the treatment at 4–24 h (*P* < 0.05). However, the patterns were inconsistent and no remarkable increase in OD260s was observed in relation to the exposure time.

### 3.5. Transmission Electron Microscopy

**Figure 4:**
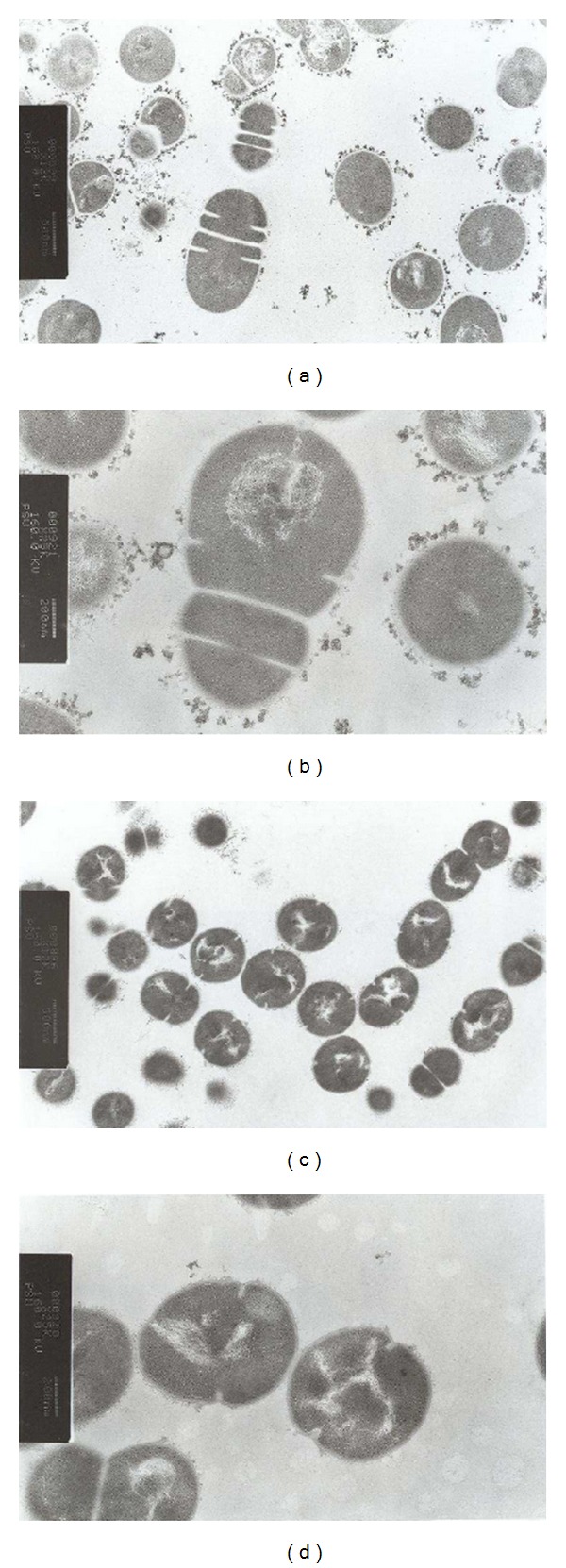
Transmission electron micrographs of the ethanol extract of *Rhodomyrtus tomentosa* treated *Streptococcus pyogenes* NPRC 101 cells at MBC concentration for 14 h according to the time before the cells were killed with the extract ((a) and (b)). 1% DMSO was used as control ((c) and (d)). Magnification: ×12000 ((a) and (c)) and ×25000 ((b) and (d)).

Transmission electron microscopy was carried out to observe any morphological changes in *S. pyogenes* cells caused by the plant extract at its MBC value (Figure 4). The bacterial cells were collected after 14 h before they were killed according to the time-kill assay (Figure 1). The extract of *R. tomentosa* did not cause any dramatic changes on the bacterial cells before they were killed. However, a few treated cells seem to have some disorders during cell division process (Figures 4(a) and 4(b)). The treated cells demonstrated irregular shape with different sizes during binary fission.

## 4. Discussion

An increase in antibiotic-resistant *S. pyogenes* and failure rates of antibiotic therapy for *S. pyogenes* infections has been described [3, 12–15, 25]. A number of medicinal plants have been reported to have anti-*S. pyogenes* activities [26]. Our results revealed that *R. tomentosa *extract produced very good antibacterial activity against all 47 clinical *S. pyogenes* isolates. Nevertheless, little is known about the antibacterial mechanism and pharmacological effects of this effective plant and its active compound. Determination of their mechanisms of action will provide a starting point for the discovery of new medicines. A previous study of 73 essential oils against *Streptococcus pneumoniae* reported that the essential oils from oregano, rosewood, and thyme caused membrane damage and induced cell lysis [27]. *Melaleuca alternifolia* (tea tree) oil and its components have been reported to induce delayed lysis in *Staphylococcus aureus* by releasing membrane-bound cell wall autolytic enzymes when the bacterial cells were treated for several hours [23]. In contrast,* R. tomentosa* extract did not cause any delayed effect, and the action of this extract does not appear to involve cell wall damage or activation of autolytic enzymes. Leakage in the cytoplasmic membrane was analysed by determining of the absorbance reading at 260 nm to detect the release of cell materials including nucleic acid, metabolites, and ions [28]. Several antibacterial agents and plant extracts such as chlorhexidine [29], hexachlorophene [30], phenethyl alcohol [31], lemongrass oil [32], tea tree oil [23], and *Eleutherine americana* extract [33] have shown an effect on bacterial cytoplasmic membranes through inducing a loss of 260-nm-absorbing materials. Our results showed slight loss of 260-nm-absorbing materials, suggesting loss of negligible amount of nucleic acids through a damaged cytoplasmic membrane. This leakage could also be caused by other reasons such as weakening of the cell wall or disruption of cell membrane by osmotic pressure when the bacterial cells were kept for several hours. As revealed in the ultrastructural analysis of the treated cells, this extract did not reveal any dramatic changes on the bacterial cells. However, a few of treated cells demonstrated irregular shape with different sizes during binary fission.

Rhodomyrtone, an isolated active compound from *R. tomentosa*, exhibited strong antibacterial activity against *S. pyogenes*. Rhodomyrtone is a newly reported novel antibacterial compound and there have been very brief reports on its activity. Significant antibacterial activities of rhodomyrtone against many Gram-positive bacteria have been documented [18, 34]. Although the previous researches have attempted to explain the actions of rhodomyrtone [35–37], its antibacterial mechanisms of action are still unclear. Therefore, the development of this compound as a new lead drug needs more information and researches on its modes of action.

The results obtained from this study shed light on the antibacterial mechanisms of *R. tomentosa* leaf extract. Rhodomyrtone has powerful *in vitro* activity against *S. pyogenes*. This active compound may be used as a therapeutic drug candidate for the control of streptococcal infections. Further research that examines it's *in vitro* and *in vivo* mechanisms of action, toxicity, and therapeutic effect is necessary.

## Figures and Tables

**Table 1:** The minimum inhibitory concentration (MIC) and minimum bactericidal concentration (MBC) of antibiotics, ethanol extract of *Rhodomyrtus tomentosa*, and rhodomyrtone against clinical *Streptococcus pyogenes* isolates.

Antibacterial agents	Antibacterial activity (*μ*g mL^−1^)						Resistance (%)
	*MIC_50_	^†^MIC_90_	MIC range	^‡^MBC_50_	^§^MBC_90_	MBC range
Antibiotics (*n* = 47)							
Erythromycin	<0.015	0.031	<0.015–0.125	0.031	0.031	<0.015–0.125	0
Penicillin G	<0.015	<0.015	<0.015–0.062	<0.015	0.031	<0.015–0.062	0
Plant extract/compound							
Ethanol extract (*n* = 47)	7.81	15.62	3.91–62.50	15.62	15.62	3.91–62.50	
Rhodomyrtone (*n* = 14)	0.78	1.56	0.39–1.56	1.56	1.56	0.39–1.56	

*MIC at which 50% of the isolates were inhibited (MIC_50_).

^†^MIC at which 90% of the isolates were inhibited (MIC_90_).

^‡^MBC at which 50% of the isolates were killed (MBC_50_).

^§^MBC at which 90% of the isolates were killed (MBC_90_).

*n*: Number of test isolate.
